# Bitter Melon (*Momordica charantia* L.) Rootstock Improves the Heat Tolerance of Cucumber by Regulating Photosynthetic and Antioxidant Defense Pathways

**DOI:** 10.3390/plants9060692

**Published:** 2020-05-29

**Authors:** Mei-Qi Tao, Mohammad Shah Jahan, Kun Hou, Sheng Shu, Yu Wang, Jin Sun, Shi-Rong Guo

**Affiliations:** 1 Key Laboratory of Southern Vegetable Crop Genetic Improvement in Ministry of Agriculture, College of Horticulture, Nanjing Agricultural University, Nanjing 210095, China; 2017104116@njau.edu.cn (M.-Q.T.); shahjahansau@gmail.com (M.S.J.); 2017104117@njau.edu.cn (K.H.); shusheng@njau.edu.cn (S.S.); ywang@njau.edu.cn (Y.W.); jinsun@njau.edu.cn (J.S.); 2 Department of Horticulture, Sher-e-Bangla Agricultural University, Dhaka 1207, Bangladesh; 3 Suqian Academy of Protected Horticulture, Nanjing Agricultural University, Suqian 223800, China

**Keywords:** heat stress, grafting, cucumber, bitter-melon rootstock, polyamines, photosynthesis

## Abstract

High temperature is considered a critical abiotic stressor that is increasing continuously, which is severely affecting plant growth and development. The use of heat-resistant rootstock grafting is a viable technique that is practiced globally to improve plant resistance towards abiotic stresses. In this experiment, we explored the efficacy of bitter melon rootstock and how it regulates photosynthesis and the antioxidant defense system to alleviate heat stress (42 °C/32 °C) in cucumber. Our results revealed that bitter-melon-grafted seedlings significantly relieved heat-induced growth inhibition and photoinhibition, maintained better photosynthesis activity, and accumulated a greater biomass than self-grafted seedlings. We measured the endogenous polyamine and hydrogen peroxide (H_2_O_2_) contents to determine the inherent mechanism responsible for these effects, and the results showed that heat stress induced a transient increase in polyamines and H_2_O_2_ in the inner courtyard of grafted seedlings. This increment was greater and more robust in bitter-melon-grafted seedlings. In addition, the use of polyamine synthesis inhibitors MGBG (methylglyoxal bis-guanylhydrazone) and D-Arg (D-arginine), further confirmed that the production of H_2_O_2_ under heat stress is mediated by the accumulation of endogenous polyamines. Moreover, compared with other treatments, the bitter-melon-grafted seedlings maintained high levels of antioxidant enzyme activity under high temperature conditions. However, these activities were significantly inhibited by polyamine synthesis inhibitors and H_2_O_2_ scavengers (dimethylthiourea, DMTU), indicating that bitter melon rootstock not only maintained better photosynthetic activity under conditions of high temperature stress but also mediated the production of H_2_O_2_ through the regulation of the high level of endogenous polyamines, thereby boosting the antioxidant defense system and comprehensively improving the heat tolerance of cucumber seedlings. Taken together, these results indicate that grafting with a resistant cultivar is a promising alternative tool for reducing stress-induced damage.

## 1. Introduction

During their life cycle, plants face various environmental stimuli, including high temperature stress. In recent years, with the rise in global temperature, the greenhouse effect has continuously increased, and high temperatures have become a major environmental threat that adversely affects crop growth and productivity [1,2,3,4,5]. In China’s facility cultivation, especially in southern China, facility cultivation is frequently subjected to high-temperature stress [6], which leads to the suppression of crop growth and seriously inhibits the production and supply of vegetables [7].

Photosynthesis is one of the most heat-sensitive biological processes [8]. The activity of photosynthesis is directly associated with the amount of biomass production in plants [9]. It has been reported in several studies that heat stress can lead to the inhibition of plant photosynthesis and is the main reason for a reduction in crop yield [5,10,11,12]. The main reason for the reduction of photosynthesis is the inhibition of photosystem II (PSII) [13]. At the same time, PSII is considered to be a key part of high-temperature stress-induced photoinhibition [7].

Recently, a couple of studies showed that polyamines play a direct controlling role in regulating resistance against different types of plant stress, such as salt stress [14,15,16], heat stress [17], low temperature stress [18], drought stress [19], and flood stress [20,21]. Polyamines participate in complex signaling systems under abiotic stress which, in turn, regulates a series of defense responses in plants, thereby improving plant resistance against different environmental stressors [22]. There is a strong link between polyamines and different signaling molecules, such as H_2_O_2_, NO, and Ca^2+^, and these signaling molecules mediate the mitigation effect of polyamines on stress [23,24,25,26,27].

Grafting is a mature technical method that is used to enhance the stress tolerance of plants [28,29,30]. Grafting roots play a vital role in plants’ response to various stressors [31]. The tolerance of grafted rootstocks to adverse conditions directly affects the resistance of grafted seedlings [29]. Some previous studies have confirmed that resistant rootstock grafting can improve the stress tolerance of grafted plants by reducing photosynthesis inhibition [32], regulating osmotic substances [7], enhancing antioxidant defense [33], regulating hormone signaling [34], and mediating with microRNA transcription [35]. Moreover, rootstock grafting can improve the salt tolerance of cucumber seedlings by regulating endogenous polyamine metabolism. However, the specific role of endogenous polyamines and their regulatory networks in grafted plants under stressful conditions, particularly high temperature stress, has still not been fully elucidated. 

Cucumber is an important facility horticultural crop with a high level of heat sensitivity. Bitter melon originated in India and is not cold-tolerant but is heat-resistant [6]. Therefore, we extensively studied the effect of heat-resistant bitter melon rootstock on the photosynthesis of grafted cucumber under conditions of high temperature stress and the physiological mechanism by which grafting alleviates the high temperature stress injury of cucumber plants. We also explored the regulatory mechanism of endogenous polyamines and H_2_O_2_ signaling molecules in grafted plants, and our results provide a theoretical basis for the cultivation of facility crops under a high-temperature regime.

## 2. Results

### 2.1. Effects of Bitter Melon Rootstock on Plant Growth under Heat Stress

**Figure 1:**
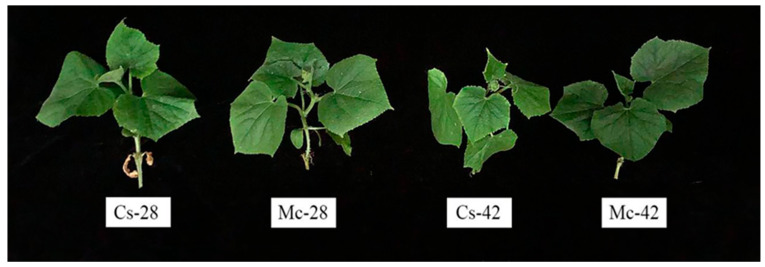
Interactive effects of bitter melon rootstock and grafted cucumber seedlings on the phenotype of cucumber seedlings after 7 days of heat stress. Self-grafted plants treated with 28 °C/18 °C (day/night), Cs-28; bitter-melon-grafted plants treated with 28 °C/18 °C (day/night), Mc-28; self-grafted plants treated with 42 °C/32 °C (day/night), Cs-42; bitter-melon-grafted plants treated with 42 °C/32 °C (day/night), Mc-42.

The growth attributes such as plant height, fresh and dry weight of self-grafted plants showed significantly greater values in control plants than in bitter-melon-grafted plants, except for the stem diameter (Table 1, Figure 1). In contrast, after 7 days of high-temperature treatment, except for the plant height, bitter-melon-grafted seedlings had significantly higher stem diameters and above-ground fresh and dry biomass weights than the self-grafted seedlings. Specifically, compared with the control, the plant height, stem thickness, and fresh and dry biomass weights of the self-grafted seedlings were reduced by 4.03%, 5.52%, 20.30%, and 29.51%, respectively, while the above four indicators were reduced by 0.25%, 4.09%, 6.26%, and 15.98%, respectively, in bitter-melon-grafted seedlings, indicating that bitter-melon-grafted seedlings could maintain greater biomass accumulation after high-temperature stress (Table 1).

### 2.2. Effects of Bitter Melon Rootstock on Photosystem II (PSII) Photochemistry under Heat Stress Conditions

**Figure 2:**
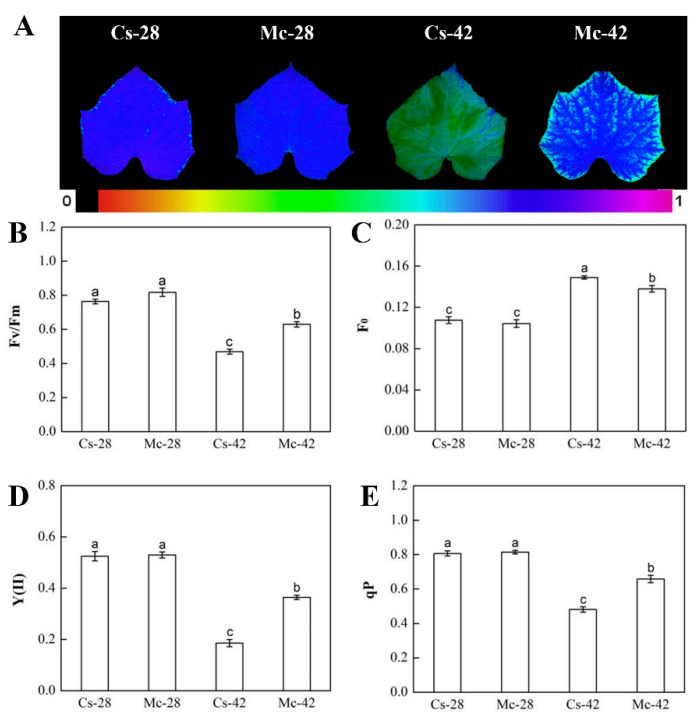
Interactive effects of bitter melon rootstock and grafted cucumber seedlings on chlorophyll fluorescence following exposure to heat stress for 7 days. The maximal photochemical efficiency of photosystem II (PSII, *Fv*/*Fm*) (**A**,**B**) under the initial fluorescence (*F*_0_) (**C**), the effective photochemical quantum yield of PSII (Y(II)) (**D**) under photochemical quenching (qP) (**E**). Bars represent the mean ± SE of at least three independent experiments. Self-grafted plants exposed to 28 °C/18 °C (day/night), Cs-28; bitter-melon-grafted plants exposed to 28 °C/18 °C (day/night), Mc-28; self-grafted plants exposed to 42 °C/32 °C (day/night), Cs-42; bitter-melon-grafted plants exposed to 42 °C/32 °C (day/night), Mc-42. Different letters indicate significant differences at *p* < 0.05 according to Duncan’s multiple range test.

To explore the protective effect of rootstock grafting on photosynthetic systems, the following photosynthetic parameters were measured. As shown in Figure 2, there were no significant differences in *Fv*/*Fm*, *F*_0_, Y(II), and qP between self-grafted plants and the bitter-melon-grafted plants under normal growth conditions. Conversely, heat stress significantly reduced the *Fv*/*Fm*, Y(II), and qP in both self-grafted and bitter-melon-grafted plants (Figure 2A,B,D,E), but the above three indicators were, respectively, 43.2%, 96.2%, and 36.8% higher in bitter-melon-grafted plants than in self-grafted plants under heat stress conditions. However, the *F*_0_ value in both grafted plants was increased under heat stress conditions (Figure 2C), and the value of *F*_0_ in the bitter-melon-grafted plant was considerably reduced compared with that of the self-grafted plants. 

### 2.3. Effect of Bitter Melon Rootstock on the Endogenous Polyamine Content under Heat Stress Conditions

**Figure 3:**
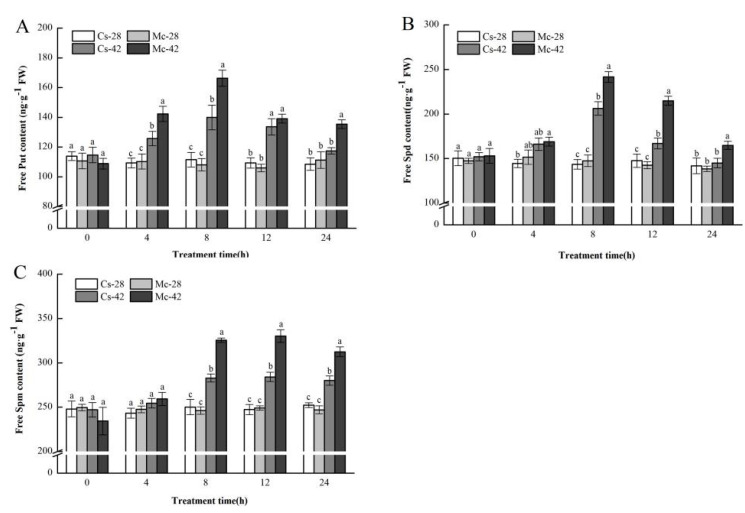
Interactive effects of bitter melon rootstock and grafted cucumber seedlings on the time course change of polyamine contents (**A**): Putrescine (Put), (**B**): Spermidine (Spd), (**C**): Spermine (Spm) in grafted cucumber leaves under heat stress conditions. The bars represent the mean ± SE values of at least three independent experiments. Self-grafted plants exposed to 28 °C/18 °C (day/night), Cs-28; bitter-melon-grafted plants exposed to 28 °C/18 °C (day/night), Mc-28; self-grafted plants exposed to 42 °C/32 °C (day/night), Cs-42; bitter-melon-grafted plants exposed to 42 °C/32 °C (day/night), Mc-42. Different letters indicate significant differences at *p* < 0.05 according to Duncan’s multiple range test.

As displayed in Figure 3, under normal conditions, the endogenous contents of the three free polyamines in the leaves of bitter-melon-grafted seedlings and self-grafted seedlings were not significantly different throughout the whole treatment period. After exposure to heat stress, the accumulation of polyamines in the leaves of the two grafted seedlings was significantly greater than the control level, and the polyamine content in the leaves of bitter-melon-grafted seedlings was significantly higher than that of self-grafted seedlings. The specific performance was as follows: the putrescine (Put) content of the two grafted seedlings began to rise at 4 h after heat stress and became significantly higher than the control level. It began to decline after reaching the peak at 8 h; at this time, the contents of spermidine (Spd) and spermine (Spm) also began to rise slightly. Spd and Spm began to decline after reaching their peak values at 8 and 12 h, respectively. It is important to note that the content of Spm at 8 h was close to that at 12 h.

### 2.4. Effect of Bitter Melon Rootstock on the Accumulation of H_2_O_2_ under Heat Stress Conditions

**Figure 4:**
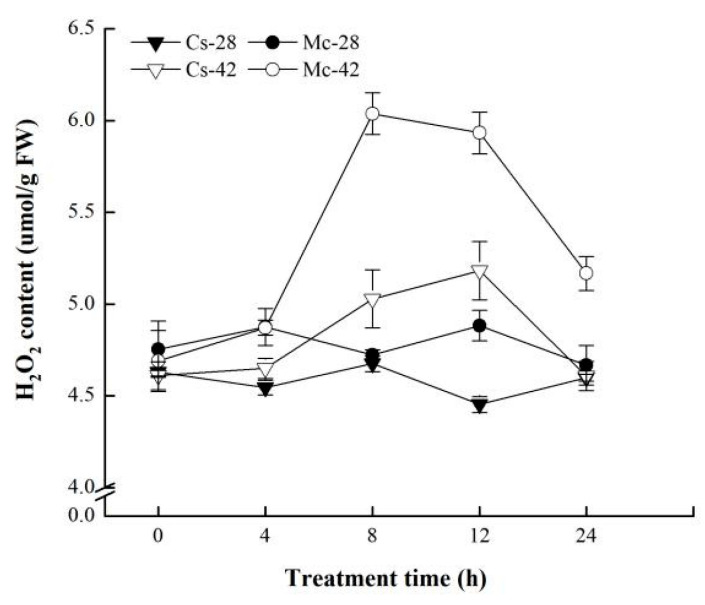
Interactive effects of bitter melon rootstock and grafted cucumber seedlings on the time course change of the H_2_O_2_ content in grafted cucumber leaves under heat stress conditions. Self-grafted plants exposed to 28 °C/18 °C (day/night), Cs-28; bitter-melon-grafted plants exposed to 28 °C/18 °C (day/night), Mc-28; self-grafted plants exposed to 42 °C/32 °C (day/night), Cs-42; bitter-melon-grafted plants exposed to 42 °C/32 °C (day/night), Mc-42. The values are the means ± SE of three independent experiments. The bars represent the standard error.

As shown in Figure 4, throughout the whole treatment period, there was no significant change in the H_2_O_2_ content in the leaves of the two grafted seedlings under control conditions; however, high temperature stress significantly increased the H_2_O_2_ content in the leaves of bitter-melon-grafted seedlings and self-grafted seedlings, to a value significantly higher than that in the control plants. Under heat stress conditions, the changing trend of the H_2_O_2_ content in the leaves of the two grafted seedlings was roughly the same, that is, the H_2_O_2_ content increased first and then decreased. However, the increase in the H_2_O_2_ content of bitter-melon-grafted seedlings was substantially higher than that of self-grafted seedlings at 8, 12, and 24 h. Under heat stress conditions, the H_2_O_2_ content of the leaves of bitter-melon-grafted seedlings increased rapidly after 4 h, and it reached a peak at 8 h, and then began to decline. The peak content was about 1.28 times more than the control level; the self-grafted seedlings also showed a slow upward trend after 4 h. Furthermore, it began to decline after reaching the peak at 12 h. The peak content was only 1.16 times higher than the control, and it returned to the control level after 24 h.

### 2.5. The Production of H_2_O_2_ is Regulated by Polyamines under Heat Stress Conditions

**Figure 5:**
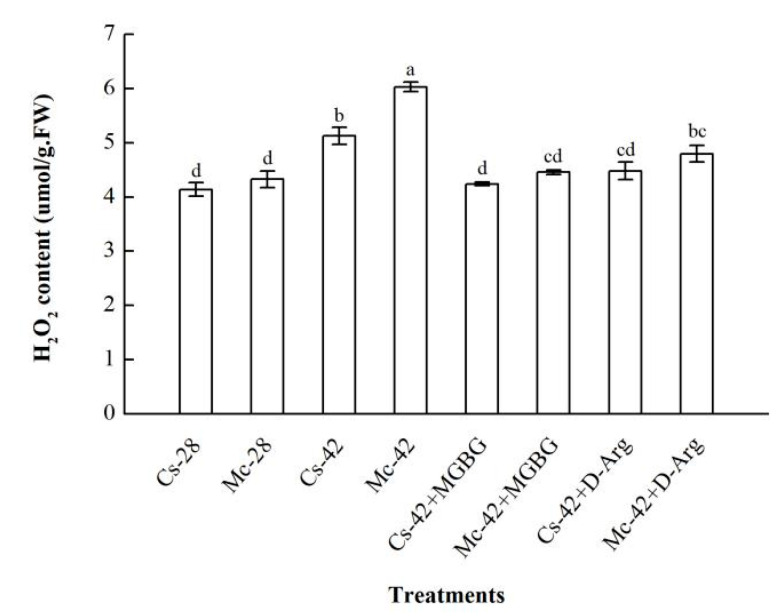
Effects of D-arginine (D-Arg) and methylglyoxal bis-guanylhydrazone (MGBG) on H_2_O_2_ production in the leaves of self-grafted and bitter-melon-grafted cucumber seedlings under heat stress conditions. Self-grafted plants exposed to 28 °C/18 °C (day/night), Cs-28; bitter-melon-grafted plants exposed to 28 °C/18 °C (day/night), Mc-28; self-grafted plants exposed to 42 °C/32 °C (day/night), Cs-42; bitter-melon-grafted plants exposed to 42 °C/32 °C (day/night), Mc-42. Seedlings were pretreated with 0.2 mM MGBG and 2mM D-Arg for 12 h, respectively, after which they were treated under high temperature conditions for 8 h. Bars represent the mean ± SE of at least three independent experiments. The different letters indicate significant differences at *p* < 0.05 according to Duncan’s multiple range test.

D-arginine (D-Arg) and methylglyoxal bis-guanylhydrazone (MGBG) are two kinds of polyamine synthesis inhibitor. To explore whether polyamines mediate H_2_O_2_ production in the leaves of grafted seedling under conditions of high temperature stress, we exogenously applied two kinds of inhibitor. As shown in Figure 5, treatment with both 1mM MGBG and 2 mM D-Arg significantly reduced the high-temperature-induced greater accumulation of H_2_O_2_ and almost returned to the control level, indicating that polyamines mediate the production of H_2_O_2_ under conditions of high temperature stress.

### 2.6. Polyamine and H_2_O_2_ Accumulation Enhanced the Antioxidant Defense System of Bitter-Melon-Grafted Seedling Leaves under Heat Stress Conditions

**Figure 6:**
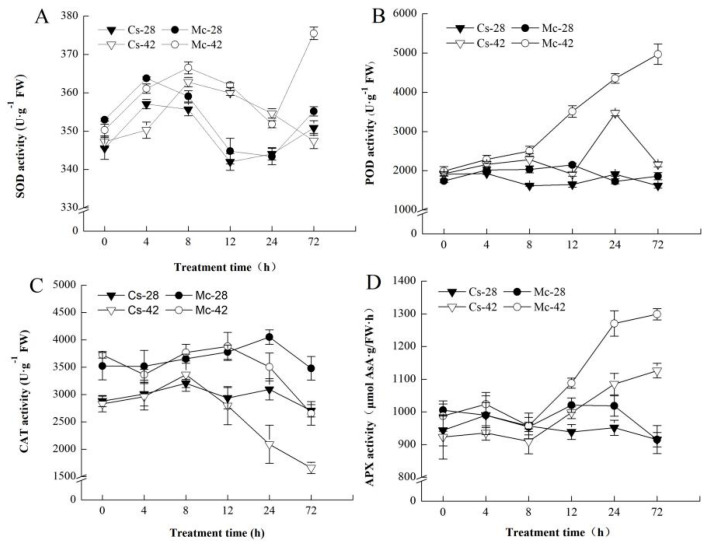
Interactive effects of bitter melon rootstock and grafted cucumber on the time course change of enzyme activity of (**A**) Superoxide dismutase (SOD), (**B**) Peroxidase (POD), (**C**) Catalase (CAT) and (**D**) Ascorbate peroxidase (APX) in cucumber leaves under heat stress conditions. Self-grafted plants exposed to 28 °C/18 °C (day/night), Cs-28; bitter-melon-grafted plants exposed to 28 °C/18 °C (day/night), Mc-28; self-grafted plants exposed to 42 °C/32 °C (day/night), Cs-42; bitter-melon-grafted plants exposed to 42 °C/32 °C (day/night), Mc-42. The values are the mean ± SE of three independent experiments. The bars represent the standard error.

As shown in Figure 6, heat stress significantly induced the activity of three antioxidant enzymes: super oxide dismutase (SOD), peroxidase (POD), and ascorbate peroxidase (APX) in grafted seedlings. In particular, the bitter-melon-grafted seedlings exhibited higher enzyme activities. The activities of SOD and POD markedly increased at 8 h after heat treatment (Figure 6A,B), while 4 h later, the activity of APX increased (Figure 6D). Interestingly, the activity of catalase (CAT) showed a clear downward trend from 24 h after heat treatment (Figure 6C). More importantly, it can be clearly seen that the peak point of the antioxidant enzyme activities was significantly lower than the polyamines (PAs) and H_2_O_2_ contents (Figure 4).

**Figure 7:**
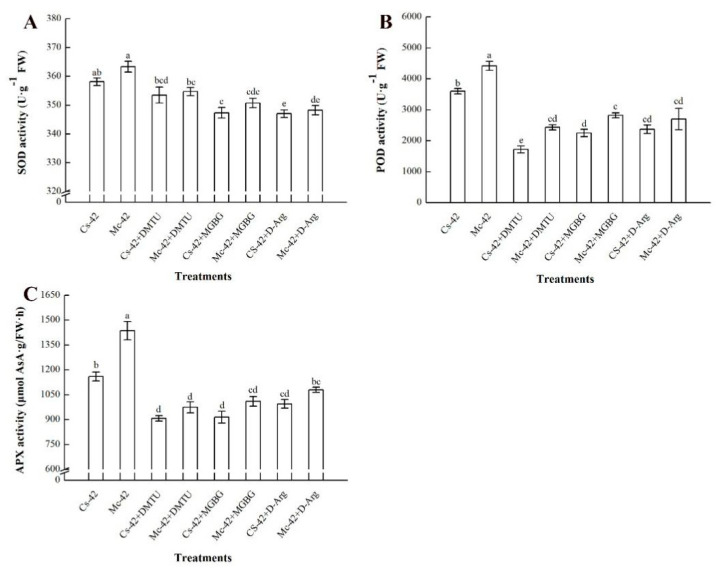
Effects of polyamine synthesis inhibitors D-arginine (D-Arg) and methylglyoxal bis-guanylhydrazone (MGBG) and an H_2_O_2_ scavenger (DMTU) on enzyme activity of (**A**) Superoxide dismutase (SOD), (**B**) Peroxidase (POD), and (**C**) Ascorbate peroxidase (APX) in grafted cucumber leaves under heat stress conditions. Self-grafted plants treated with 42 °C/32 °C (day/night), Cs-42; bitter-melon-grafted plants treated with 42 °C/32 °C (day/night), Mc-42. All inhibitors were sprayed 12 h before exposure to heat stress. Seedlings were treated for 24 h under heat stress conditions. Bars represent the mean ± SE of at least three independent experiments. The different letters indicate significant differences at *p* < 0.05 according to Duncan’s multiple range test.

To further elucidate how PAs and H_2_O_2_ contributed to the change in antioxidant enzyme activities under conditions of high temperature stress, polyamine synthesis inhibitors (MGBG and D-Arg) and hydrogen peroxide scavengers (DMTU) were applied in subsequent experiments. The results are shown in Figure 7. The activity of the three enzymes in the grafted plants sprayed with the three inhibitors was markedly lower than that of the grafted plants under only heat stress conditions. Combined with previous results (Figure 5), it is speculated that polyamines may mediate the production of hydrogen peroxide and further regulate the antioxidant defense.

## 3. Discussion

In the last few decades, high-temperature stress has become a major environmental stressor that restricts proper growth and yield of crops [36,37], and the promotion of grafting technology provides an opportunity to improve crop resistance to various biotic and abiotic stresses, including high-temperature stress [6]. In our study, heat stress at 42/32 °C (day/night) significantly prevented the growth of grafted seedlings. However, seedlings grafted with bitter melon rootstock showed higher stem thicknesses and above-ground dry/fresh weights than self-grafted seedlings under conditions of high-temperature stress, showing that bitter melon rootstock efficiently ameliorated the growth inhibition caused by heat stress (Table 1, and Figure 1). Similar to the bitter melon rootstock, the grafting of heat-resistant luffa rootstock also alleviated the growth inhibition of cucumber seedlings caused by heat stress [33]. Moreover, in addition to high-temperature stress, rootstock grafting has also shown a mitigating effect on growth inhibition under exposure to other abiotic stresses [38,39]. Leaf photosynthesis performs a vital function in determining crop yield [12], and electron transfer during photosynthesis is very sensitive to heat stress, which is the main limiting factor of photosynthesis under high-temperature stress [40]. PSII is also considered to be more vulnerable to high-temperature damage [7]. Our results indicate that high temperatures induce photoinhibition of grafted seedlings, which is manifested in decreases in *Fv*/*Fm*, qP, and Y (II) and an increase in *F*_0_. However, bitter melon rootstock significantly alleviated the photoinhibition caused by high-temperature stress. The changes in fluorescence parameters agreed with previous results of studies where cucumbers were grafted onto other rootstocks under heat stress conditions [32,41].

Polyamines have been widely reported to have an important role in the plant response to abiotic stress [42,43,44]. In our study, the results showed that high temperatures induce a large and rapid increase in the free endogenous PAs contents in the leaves of grafted seedlings, which mostly reached a peak at 8 h (Figure 3). The trend was identical to the time course of the changes in the endogenous PA level in cucumber plants under salt stress [23]. In addition, the increased content of PAs in the leaves of bitter-melon-grafted seedlings was more prominent than that in self-grafted seedlings. All of these results imply that rootstock grafting may produce a stronger response to stress by increasing endogenous polyamine levels.

With the deepening of research, more studies have indicated that polyamines participate in the regulation of complex signal systems to resist stress [22,24,26]. Hydrogen peroxide has also been frequently documented as a signaling molecule that plays a role in different stresses [45,46,47]. Moreover, in recent years, studies have confirmed that there are interconnections between polyamine and hydrogen peroxide signaling molecules under stress conditions [48,49]. However, it is unclear whether they respond similarly to grafted plants exposed to high-temperature stress. In the present study, our results showed that high-temperature stress induces a rapid response from H_2_O_2_ (Figure 4), and the changing trend of the content of H_2_O_2_ is consistent with the results of our previous study [50]. Moreover, grafted seedlings showed an obvious sequence in response to H_2_O_2_, whereby the bitter-melon-grafted seedlings peaked at 8 h, while the self-grafted seedlings peaked at 12 h, indicating that bitter-melon-grafted seedlings can accumulate hydrogen peroxide more quickly and significantly in response to high-temperature stress. To further explore the correlation between PAs and H_2_O_2_, starting from the difference between the peaks of PAs and H_2_O_2_ in the leaves of self-grafted cucumber seedlings, polyamine synthesis inhibitors were used to investigate whether changes in endogenous polyamines under stress have a mediating effect on H_2_O_2_. The results showed (Figure 5) that pretreatment with D-Arg and MGBG dramatically decreased the H_2_O_2_ content in the leaves of grafted seedlings under conditions of heat stress treatment. Therefore, it is speculated that the H_2_O_2_ content in the leaves is mediated by endogenous polyamines. The result is consistent with findings in cucumber seedlings under salt stress [23].

The antioxidant defense system is a key strategy by which plants cope with abiotic stress [4,5,51,52]. Some previous reports have shown that polyamines and H_2_O_2_ signaling molecules actively participate in antioxidant defense under exposure to a stressful environment [23,26]. In this study, under conditions of high temperature stress, bitter-melon-grafted seedlings maintained a high level of antioxidant enzyme activity (except CAT) (Figure 6), similar to that observed in wheat seedling leaves under a heat stress environment [53], and the peak of antioxidant enzyme activity occurred after the production of PAs and H_2_O_2_. From this, we speculate that the heat-induced antioxidant defense in bitter-melon-grafted seedlings is triggered by polyamines and involves H_2_O_2_. In order to verify our conjecture, a follow-up inhibitor test was designed. Our result showed that the polyamine synthesis inhibitors (D-Arg and MGBG) and H_2_O_2_ scavenger (DMTU) significantly reduced the higher level of antioxidants in the leaves of rootstock-grafted seedlings under conditions of high-temperature stress (Figure 7). This result is very similar to that of exogenous polyamines, and this was confirmed by increasing the content of endogenous polyamines to regulate the hydrogen peroxide signal, thereby improving the antioxidant defense capacity of cucumber seedlings under salt stress [24]. It further shows that under a high-temperature stress environment, both polyamines and H_2_O_2_ mediate the antioxidant defense of grafted seedlings. Under heat stress conditions, the bitter-melon-grafted seedlings showed greater endogenous polyamine accumulation and a more sensitive H_2_O_2_ response than the self-rooted grafted seedlings. Thus, they exhibited a stronger antioxidant defense capacity, and the damage to plants caused by heat stress was significantly attenuated.

## 4. Materials and Methods 

### 4.1. Plant Materials and Treatments

Cucumber (*Cucumis sativus* L., cv. Jinyou No.35) was used as the scion (Cs), and bitter melon (*Momordica Charantia* L., cv. Changlv) was used as the rootstock (Mc). In this study, cleft grafting was used for grafting and self-grafted plants were used as a control. Uniform and germinated seeds of bitter melon were sown in organic substrates (2:2:1 [*v*/*v*/*v*] vinegar waste compost/peat/vermiculite; Peilei, Zhenjiang, China). When the cotyledons of cucumber rootstock were flattened, and bitter melon rootstock was outcropped, the cucumber scion was sown into a vermiculite-plated plastic square dish. When the cotyledons of the scions and the second true leaves of the rootstock were fully expanded, cleft grafting was performed. Grafted plants were transferred to a small, plastic, arched shed, where the environment was maintained as follows: a temperature of around 25 °C and a relative humidity of 85–100% for 7 days until the graft union had completely healed. After the third true leaves were fully expanded, grafted plants were shifted to a growth chamber (RDN-560E-4; Dongnan Instrument, Ningbo, China), where a photosynthetic photon flux density (PPFD) of 300 μmol m^−2^ s^−1^, a relative humidity of 70–75%, and a 12/12 h (day/night) light/dark photoperiod were maintained.

When seedlings attained the fourth leaves stage, the plants underwent different treatments: (1) self-grafted plants were exposed to 28 °C/18 °C (day/night), Cs-28; (2) bitter-melon-grafted plants were exposed to 28 °C/18 °C (day/night), Mc-28; (3) self-grafted plants were exposed to 42 °C/32 °C (day/night), Cs-42; (4) bitter-melon-grafted plants were exposed to 42 °C/32 °C (day/night), Mc-42; (5) self-grafted plants were pretreated with 0.2 mM methylglyoxal bis-guanylhydrazone (MGBG, an inhibitor of SAMDC-S-adenosyl methionine decarboxylase) and then exposed to 42 °C/32 °C (day/night), Cs-42 + MGBG; (6) bitter-melon-grafted plants were pretreated with 1 mM methylglyoxal bis-guanylhydrazone (MGBG, an inhibitor of SAMDC) and then exposed to 42 °C/32 °C (day/night), Mc-42+MGBG; (7) self-grafted plants were pretreated with 2 mM D-Arginine (D-Arg, an inhibitor of ADC) and then exposed to 42 °C/32 °C (day/night), Cs-42 + D-Arg; (8) bitter-melon-grafted plants were pretreated with 2 mM D-Arginine (D-Arg, an inhibitor of ADC), and then exposed to 42 °C/32 °C (day/night), Mc-42 + D-Arg; (9) self-grafted plants were pretreated with 5 mM dimethylthiourea (DMTU, a H_2_O_2_ scavenger) and then exposed to 42 °C/32 °C (day/night), Cs-42 + DMTU; (10) bitter-melon-grafted plants were pretreated with 5 mM dimethylthiourea (DMTU, a H_2_O_2_ scavenger) and then exposed to 42 °C/32 °C (day/night), Mc-42 + DMTU. The plants were sprayed with inhibitors or scavengers 12 h before the high-temperature treatment. The leaves sampled were collected after different treatment time points (for the durations of the different chemical treatments, please see each figure legend) and stored at −80 °C until subsequent analysis.

### 4.2. Measurement of Plant Growth

The distance between the graft junctions to the scion growth point was measured by a ruler and taken to be the plant height. The thickness of the scion stem in the direction parallel to the scion cotyledon was determined by an electronic Vernier caliper, and this represented the stem thickness. To determine the fresh weight of the above-ground plant parts, we first washed them with distilled water and then wiped off excess water and measured the fresh weight. After drying at 115 °C for 15 min, the above-ground plant parts were then oven-dried at 75 °C to obtain their constant dry weights.

### 4.3. Measurement of Chlorophyll Fluorescence

Chlorophyll fluorescence measurement was undertaken with the method described by [54] using the M series chlorophyll fluorescence imaging system (Walz, Effeltrich, Germany), and Imaging-Win software was used to obtain the fluorescence parameter data and image. 

### 4.4. Quantification of Endogenous Polyamines

Free polyamines were determined using the method of [55] with a few modifications. Firstly, for polyamine extraction, 0.5 g of leaves was homogenized with 1.6 mL of cold 5% (*w*/*v*) perchloric acid (PCA) in an ice bath for 1 h followed by centrifugation at 12,000× *g* for 20 min at 4 °C. The supernatant was then used to determine the free polyamine content. To 0.7 mL of supernatant, 1.4 mL of NaOH (2M) and 15 μL of benzoyl chloride were added, and the solution was vortexed for 30 s, after which it was incubated for 30 min at 37 °C. After that, to terminate the reaction, 2 mL of saturated NaCl solution was added to the resulting solution. To extract benzoyl polyamines, 2 mL of diethyl ether was added to the mixed solution, followed by centrifugation at 3000× *g* for 5 min at 4 °C. Finally, the extracted benzoyl PAs were evaporated to dryness and then re-dissolved in 1 mL of 64% (*v*/*v*) methanol. After passing through a 0.45 μmol filter, they were stored at −20 °C. Ultra-performance liquid chromatography (UPLC) was used to determine the content of polyamines. 

### 4.5. Determination of H_2_O_2_ Content

The H_2_O_2_ concentration was determined using the method described by [56] with slight modifications. Firstly, the harvested leaf samples were homogenized in 1.6 mL of 0.1% trichloroacetic acid (TCA) followed by centrifugation at 12,000× *g* for 20 min at 4 °C. Then, 0.25 mL of 0.1 M potassium phosphate buffer (pH 7.8) and 1 mL of 1 M KI (potassium iodine) were incorporated into 0.2 mL of supernatant and held in a dark place for 1 h. After the reaction was completed, 0.1% of TCA was used as a blank control to zero, and the absorbance was read at 390 nm. Finally, the H_2_O_2_ content was estimated from a standard curve of known concentrations of H_2_O_2_.

### 4.6. Assays of Antioxidant Enzyme Activity

Two hundred milligrams of fresh leaf samples were digested in 1.6 mL of 0.05 M pre-cold phosphate buffer (pH 7.8), followed by centrifugation at 12,000× *g* for 20 min at 4 °C to obtain the supernatant. The extracted supernatant was used to assay the following antioxidant enzyme activities. 

For the estimation of superoxide dismutase (SOD) activity, we used the method developed by [57]. Forty microliters (40 μL) of supernatant were added to a reaction mixture consisting of 14.5 mM methionine, 0.05 M phosphate buffer (pH 7.8), 30 μM EDTA-Na_2_ (Disodium ethylene diamine tetra acetate dihydrate) solution, 2.25 mM nitro blue tetrazolium NBT solution, and 60 μM riboflavin solution. The SOD activity absorbance was measured at 560 nm.

For the measurement of peroxidase (POD) activity, the procedure described by [58] was used with slight modifications. In short, 40 μL of enzyme extract was incorporated into a reaction mixture consisting of 0.2 M phosphate buffer (pH 6.0), 30% H_2_O_2_ solution, and 50 mM guaiacol, and the absorbance was recorded at 470 nm.

Catalase (CAT) activity was determined by [59]. Briefly, 0.1 mL of enzyme extract was mixed with a reaction solution that contained 0.15 M phosphate buffer (pH 7.0) and 30% H_2_O_2_ solution. Measurement of the change in absorbance was conducted within 40 s at 240 nm.

For the estimation of ascorbate peroxidase (APX) activity, the method developed in [60] was used. In short, 0.1 mL of enzyme extract was mixed in a complex mixture made up of 50 mM phosphate buffer (pH 6.0), 0.1 mM EDTA–Na_2_, 5 mM AsA, and 20 mM H_2_O_2_ solution. The measurement of the change of absorbance was conducted within 40 s at 290 nm.

### 4.7. Statistical Analysis

For each measurement, at least three (3) independent biological replicates were tested. All data were statistically analyzed with the SPSS 20.0 software program (SPSS, Chicago, IL, USA) using Duncan’s multiple range test at the *p* < 0.05 level of significance.

## 5. Conclusions

In summary, our findings suggest that bitter melon rootstock improves the heat resistance of grafted seedlings by alleviating the photoinhibition induced by heat stress and improving the antioxidant defense capacity of leaves by regulating the changes in endogenous polyamines and H_2_O_2_ in leaves under conditions of high temperature stress. However, further study is needed to determine how the contents of polyamines and hydrogen peroxide act on the antioxidant defense system.

## Figures and Tables

**Table 1:** Interactive effects of bitter melon rootstock on the growth of grafted cucumber seedlings after 7 days of high temperature stress.

Treatments	Plant Height (cm)	Stem Diameter (mm)	Fresh Weight (Plant g^−1^)	Dry Weight (Plant g^−1^)
Cs-28	18.37 ± 0.32 ^a^	5.03 ± 0.13 ^bc^	17.19 ± 0.01 ^a^	1.83 ± 0.03 ^a^
Mc-28	16.27 ± 0.12 ^b^	5.62 ± 0.14 ^a^	16.62 ± 0.15 ^b^	1.69 ± 0.01 ^b^
Cs-42	17.63 ± 0.23 ^a^	4.79 ± 0.12 ^c^	13.70 ± 0.21 ^d^	1.29 ± 0.04 ^d^
Mc-42	16.23 ± 0.54 ^b^	5.39 ± 0.09 ^ab^	15.58 ± 0.21 ^c^	1.42 ± 0.02 ^c^

Note. Self-grafted plants treated with 28 °C/18 °C (day/night), Cs-28; bitter-melon-grafted plants treated with 28 °C/18 °C (day/night), Mc-28; self-grafted plants treated with 42 °C/32 °C (day/night), Cs-42; bitter-melon-grafted plants treated with 42 °C/32 °C (day/night), Mc-42. Data are the mean ± standard error (SE) of three independent experiments. Different letters indicate significant differences at *p* < 0.05 according to Duncan’s multiple range test.
